# Local weather and body condition influence habitat use and movements on land of molting female southern elephant seals (*Mirounga leonina*)

**DOI:** 10.1002/ece3.4049

**Published:** 2018-05-20

**Authors:** Laureline L. Chaise, Iris Prinet, Camille Toscani, Susan L. Gallon, William Paterson, Dominic J. McCafferty, Marc Théry, André Ancel, Caroline Gilbert

**Affiliations:** ^1^ UMR 7179 CNRS/MNHN Brunoy France; ^2^ Ecole Nationale Vétérinaire d'Alfort, Ethologie Maisons‐Alfort France; ^3^ Scottish Centre for Ecology and the Natural Environment Institute of Biodiversity Animal Health and Comparative Medicine MVLS University of Glasgow Glasgow UK; ^4^ Sea Mammal Research Unit Scottish Oceans Institute University of St Andrews St Andrews, Fife UK; ^5^ Université de Strasbourg CNRS IPHC UMR 7178 Strasbourg France

**Keywords:** body mass, GPS loggers, habitat selection, marine mammals, meteorological conditions, molt, pinnipeds, population counts

## Abstract

Southern elephant seals (*Mirounga leonina*) are known to move and aggregate while molting, but little is known about their behavior on land during this time. In this study, 60 adult females were monitored (23 with GPS tags) during four molting seasons, between 2012 and 2016 at Kerguelen Archipelago, Indian Ocean. Population surveys were recorded each year (*N* = 230 daily counts), and habitat use was analyzed in relation to the stage of the molt and local weather. Based on stage of molt, habitat use, and movements on land, we classified the molt of elephant seals into three phases: (1) a “search phase” at the initial stage of molt when grass and wallow habitats were used and characterized by greater mean distances travelled on land per day compared with the two other phases; (2) a “resident phase”: during initial and mid‐stage of molt when animals were found in grass and wallow habitats but with less distance moved on land; and (3) a “termination phase” at the final stage of molt where grass and beach habitats were occupied with no change in distances. Windchill and solar radiation influenced individual distances moved per day (mean 590 ± 237.0 m) at the mid‐ and final stage of molt such that animals travelled greater distances on days of low windchill or high solar radiation. Individual variation in distance moved and relative habitat use were also linked to body mass index (BMI) at arrival on the colony, as females with higher BMI moved less and preferred beach habitat. Moreover, the individual rate of molt increased with the use of wallows. Aggregation rate tended to be negatively correlated with distances moved. We therefore suggest that individuals face an energetic trade‐off while molting, balancing energy expenditure between movement and thermoregulation.

## 1 INTRODUCTION

Pinnipeds are highly adapted for an aquatic existence, but most species spend several weeks ashore each year for reproduction and molting (Le Boeuf & Laws, 1994; Williams & Worhty, 2002). Indeed, seasonal molting is characterized by hair renewal and results in an increase in haul‐out frequency and duration (Ling, 1970). Molt duration varies among phocid seals as some species (e.g., elephant seals and monk seals) experience a “catastrophic” molt, replacing not only their hair but also the top layer of their epidermis (Parsons, Bauer, McCafferty, Simmonds, & Wright, 2013; Yochem & Stewart, 2009). Furthermore, these species are known to fast during the molt when ashore for several weeks (Kenyon & Rice, 1959; Ling, 1968).

Molting is a highly energy demanding phase as adult female elephant seals experience on average a mass loss of 5 kg/day (Boyd, Arnbom, & Fedak, 1993; Carlini, Marquez, Daneri, & Poljak, 1999; Hindell, Slip, & Burton, 1994). As they are fasting on land, elephant seals rely on their body reserves in the form of blubber (Boyd et al., 1993; Liwanag, Berta, Costa, Budge, & Williams, 2012; Worthy, Morris, Costa, & Le Boeuf, 1992). Moreover, blubber also has an important role as insulation against cold (White & Odell, 1971; Whittow, 1987). Molting elephant seals are exposed to different environmental constraints in the colony, resulting in heat exchange with their surroundings (Hind & Gurney, 1998; Schmidt‐Nielsen, 1997). In particular, when seals haul‐out, they use thermal windows on their trunk and flippers to dissipate heat (i.e., heat dissipation; Mauck, Bilgmann, Jones, Eysel, & Dehnhardt, 2003). During the molt, increased perfusion of peripheral tissues promotes hair growth and renewal of the epidermis (Ashwell‐Erickson, Fay, Elsner, & Wartzok, 1986). This vasodilation bypasses the insulating blubber layer, increasing heat loss. Paterson et al. (2012) showed that heat losses of harbor seals vary when hauled out and reach a maximum corresponding to the peak stage of the molt. In order to facilitate rapid hair growth, elephant seals must maintain a relatively high peripheral temperature through perfusion of the skin surface and avoid vasoconstriction in response to environmental conditions. However, physiological mechanisms (such as vasomotor control of heat flow across the skin) can be supported by behavioral strategies to maintain thermal equilibrium (Heath, McGinnis, & Alcorn, 1976; White & Odell, 1971). Any energy‐saving strategy used to minimize heat loss during molting would reduce the depletion of energy reserves in blubber, with possible fitness consequences. Indeed, previous studies on pinnipeds have shown that reproductive success is linked with higher body mass or better body condition in females (South African fur seals *Arctocephalus pusillus*: Guinet, Roux, Bonnet, & Mison, 1998; northern elephant seal *Mirounga angustirostris*: Crocker, Williams, Costa, & Le Boeuf, 2001).

Thermoregulatory behaviors of seals while hauled‐out mostly involve heat dissipation (sand flipping, migration to/from the sea) in warm weather in temperate regions or in the tropics (Hawaiian monk seal *Monachus schauinslandi*: Whittow, 1987; northern elephant seal: White & Odell, 1971; Codde, Allen, Houser, & Crocker, 2016; gray seal *Halichoerus grypus*: Redman, Pomeroy, & Twiss, 2001). In phocids, some studies found a correlation between local weather conditions and haul‐out behavior in polar or temperate environments (harbor seal *Phoca vitulina*: Pauli & Terhune, 1987; Watts, 1992; Grellier, Thompson, & Corpe, 1996). Mainly solar radiation, air temperature, and wind speed influenced haul‐out numbers, while in other studies, there was no consistent effect of meteorological parameters. In elephant seals, behavior on land and physical activity was linked to weather conditions (northern elephant seal: Norris, Houser, & Crocker, 2010; southern elephant seal *Mirounga leonina*: Cruwys & Davis, 1994, 1995). In general, during the molt, elephant seals often aggregate in wallows (mud pools), when the nature of the colony's substrate allows their formation (Boyd et al., 1993; Carlini et al., 1999). Boyd et al. (1993) described the molt in two phases, with a “wallow phase” while elephant seals lose their old fur and skin, and a “shore phase” closer to the sea, when individuals have lost their old skin and are renewing their hair. However, movement patterns, distances travelled, and habitat use on land during the molt have not yet been extensively studied in this species.

The aim of this study was to investigate how the behavior of southern elephant seals on land may be driven by molt stage, environmental conditions, and body reserves at their arrival on land. Specifically, we hypothesize that movements and habitat selection are influenced by stage of molt and local weather at a group and individual level, and by body condition (i.e., BMI: body mass index) at an individual level. We propose that different types of habitat (based on substrate type: grass, wallows, beach) offer different thermal or physical properties and therefore influence habitat use. In particular, we expect that for elephant seals, the peak of the molt would be characterized by the use of wallows and limited movement. In comparison, we hypothesize greater movements when weather is poor or for individuals with lower BMI.

## 2 MATERIALS AND METHODS

### 2.1 Study site

**Figure 1:**
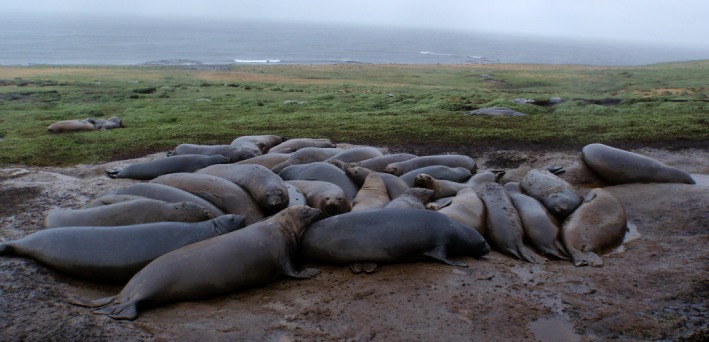
Aggregation of molting female southern elephant seals (*Mirounga leonina*) in a wallow

**Figure 2:**
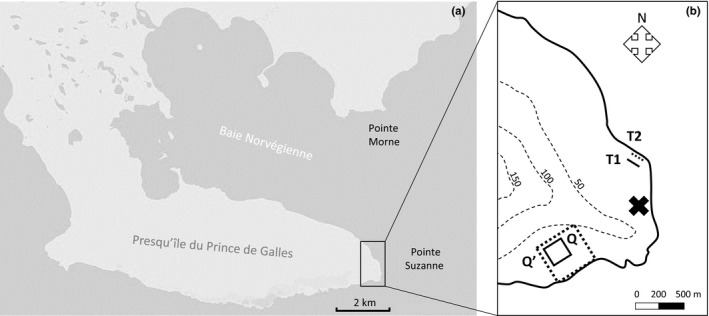
Location of Pointe Suzanne and Pointe Morne in eastern Kerguelen (a) and topography of the study site (b) with contours (m) in dotted gray lines and position of the automatic weather station (cross). Transects T1 (grass, full line) and T2 (beach, dotted line) are in the northern coast (b). Quadrats Q (wallows, full square) and Q′ (3 habitats, dotted square) are in the southern coast (b)

Data were collected during the molting season of adult females (Figure 1) from end of December to early March (Table 1) in 2012, 2014, 2015, and 2016 in the colony of Pointe Suzanne (49°26′S 70°26′E, Figure 2a) of Kerguelen Archipelago (French Southern and Antarctic Lands).

**Table 1:** Dates when counts were undertaken and the number of female elephant seals equipped with GPS loggers, along with the mean number of days of observation

Date	Transects (days)	Quadrats (days)		Good weather (days)	Bad weather (days)	Females captured [equipped with GPS]	Observation (days)
		Q	Q′				
9 January to 23 February 2012	30	14	/	/	/	15 [7]	5.87 ± 3.31
23 December 2013 to 3 March 2014	40	33	16	5	7	26 [11]	5.04 ± 2.97
24 December 2014 to 14 January 2015	11	12	11	8	2	7 [2]	1.29 ± 0.49
23 January to 26 February 2016	18	18	18	5	9	12 [3]	4.5 ± 1.57

The site is composed of a coast of stones (basalt; Nicolaysen, Frey, Hodges, Weis, & Giret, 2000), sometimes covered with seaweeds (*Durvillaea antarctica*; Lawrence, 1986), overhung by hills of grass (mainly endemic *Azorella selago* and *Acaena magellanica*, with invasive *Taraxacum officinale*; Chapuis, Frenot, & Lebouvier, 2004) where wallows (mud pools without vegetation) are formed from seal aggregations. We divided the study site into three habitats based on substrate type: “beach,” “grass,” and “wallows.” Based on the hypothesis that wallows are a specific habitat for molting, habitats “grass” and “beach” were grouped together to distinguish between “wallow” and “nonwallow” habitats for further analysis of habitat use.

### 2.2 Population surveys

To study habitat use of females during the molt, two line transects on “nonwallow” habitats (136 × 10 m; T1: on habitat “grass” and T2: on habitat “beach”) and one quadrat Q on “wallows” (200 × 200 m, Figure 2b) were selected and scanned daily (Table 1). During each scan, the total number of adult female elephant seals present was recorded, along with their molt stage assessed by the percentage (± 10%) of old hair surface shed (0%: no old hair shed to 100%: all old hair shed). We defined three stages of molt: “initial stage” (0–40% of old hair shed; still largely covered with old hair), “mid‐stage” (50–80%; mainly bare skin exposed to air with old hair shed but new hair still ungrown), and “final stage” (90–100%; new hair growing).

From 2014, a second quadrat Q′ was added (500 × 500 m) which included all three habitats (Figure 2b) to count the number of elephant seals present daily on each habitat type (Table 1) and allowed the density of female elephant seals to be monitored in detail during the molt. We thereby defined a molt season into three categories (see Results below): early season (increasing number of individuals on the colony), peak of the season (maximal number of individuals on the colony), and late season (decreasing number of individuals on the colony).

Meteorological variables including air temperature (°C), relative humidity (%), wind speed (m/s), and solar radiation (W/m²) were measured at the start of each transect and quadrat with handheld devices (Kestrel 3000 Pocket Weather Meter; pyranometer SKS111, Skye Instruments Ltd, Llandrindod Wells, UK).

### 2.3 Individual data collection

Females were captured (Table 1) and anaesthetized (Chaise et al., 2017; McMahon, Burton, Slip, McLean, & Bester, 2000) on the colony at initial stage of molt. They were recaptured at the final stage of molt, when all old hair was shed.

Females were identified, weighed (body mass; HST Mini‐Weigher; 0–1000 kg ± 0.5 kg; HST Scales UK Ltd, Milton Keynes, UK), and measured (nose–tail length ± 1 cm; 5 m tape measure) at capture and recapture to calculate initial (BMIi) and final (BMIf) body mass index (BMI = body mass (kg)/body length² (m)) and body mass loss (kg/day). Animals were equipped with VHF transmitters (Series MM300 Marine Mammal Headmount; model MM340B; 7.1 × 3.5 × 2.1 cm; 92 g; ATS Advanced Telemetry Systems, Isanti, MN, USA) to find them. Stage of molt (%), habitat use, aggregation behavior (when at least two elephant seals have physical contact), and GPS coordinates (handheld GPS eTrex^®^ 30; Garmin, Nanterre, France) were also recorded. From these observations, we calculated molt rate (%/day) as the percentage of old hair surface shed per day between first capture and first observation at 100% of old hair shed, rate of habitat use (number of observations on one habitat/total number of observations), and aggregation rate (number of observations in aggregation/total number of observations) per individual.

GPS loggers (CatLog/CatTrack1^®^; 4.7 × 3.0 × 1.3 cm; 21 g; accuracy ± 5–10 m; Catnip Technologies, Larnaca, Cyprus) were fixed on the head with epoxy bicomposed glue (Araldite^®^; Hindell, Burton, & Slip, 1991; Boyd & Arnbom, 1991) to record movements on land (Table 1).

An automatic weather station (MiniMet, Skye Instruments Ltd, Figure 2b) recorded air temperature (°C), relative humidity (%), wind speed (m/s), solar radiation (Wm^−^²), and precipitation (mm) every 30 min.

### 2.4 GPS locations

CatTracks were set up to record latitude and longitude every 30 min. Distance travelled (m) and speed (m/h) were calculated from GPS coordinates and time (GMT) of recorded positions. Mean distances per day were calculated excluding data from the day of capture and the day of recapture, to remove bias from anesthesia and capture. We used a frequency distribution of calculated speeds to select GPS reliable measures and exclude outlier positions (Ropert‐Coudert, Kato, Grémillet, & Crenner, 2012). For approximately 98.2% of recorded measures, speed was < 300 m/h. From our data, we hence considered that an elephant seal usually does not move on land at a speed higher than 300 m/h. However, based on minimum swimming speed recorded (southern elephant seals: Hindell, Lea, Morrice, & McMahon, 2000; northern elephant seals: Davis, Fuiman, Williams, & Le Boeuf, 2001), we considered that points with speed > 1000 m/h could possibly correspond to movements at sea. Therefore, measures when speed was > 300 m/h and < 1000 m/h were considered to be due to position errors (i.e., 1.1% of data). Moreover, for movements with speed > 1000 m/h, GPS coordinates of departure and arrival were mapped (ArcGIS^®^ 10, ESRI) to check whether the track was associated with travel at sea. Hence, 0.7% of tracks were considered to be at sea movements and 98.2% of data were considered to be land movements.

### 2.5 Weather data analysis

Given that weather data comprised multiple potentially colinear variables, we used a centered‐scale principal component analysis (PCA), to account for variation in meteorological parameters (air temperature, relative humidity, solar radiation, and wind speed). To examine population differences in habitat use, we used meteorological data from Q′ counts (*N* = 36, Q′ counts with complete meteorological records). The first component (PC1) accounted for 45% of the variation and the second component (PC2) accounted for 31% of the variation. We retained “day” as a coordinate on the first two axes of the PCA. PC1 received a major positive loading from solar radiation and a major negative loading from relative humidity. PC2 received a major positive loading from air temperature and a major negative loading from wind speed (Supporting Information). The components were then transformed to binary factors, corresponding to a weather index, based on their respective median values. We distinguished days of “bad weather” (for days with components value < components median value; high relative humidity, low solar radiation, low air temperature, and high wind speed) from days of “good weather” (for days with components value > components median value; low relative humidity, high solar radiation, high air temperature, and low wind speed).

To examine individual differences in distance moved during the molt, we selected meteorological parameters from the automatic weather station (air temperature, relative humidity, wind speed, solar radiation, and precipitation). The windchill index was calculated from the following equation, where windchill index = 13.12 + 0.6215 Ta + (0.3965 Ta – 11.37) × Wind^0.16^, for Ta < 10°C and Wind > 4.8 km/h (Environment and Climate Change Canada, Government of Canada; NOAA's National Weather Service, USA). Relative humidity and precipitation were positively correlated (nonparametric Spearman's rank correlation: *r* = .52, *S* = 16,614, *p *<* *.0001), and solar radiation was negatively correlated to both relative humidity and precipitation (nonparametric Spearman's rank correlation: *r* = −.50, *S* = 51,164, *p *<* *.0001; *r* = −.50, *S* = 51,477, *p *<* *.0001). To analyze variation in individual distance moved, we therefore chose windchill (i.e., the perceived decrease in air temperature felt by an endothermic organism when exposed to wind) and solar radiation (strongly influencing heat gain; Schmidt‐Nielsen, 1997) as the most important explanatory weather variables in a cold environment.

### 2.6 Statistical analysis

Generalized linear mixed models (GLMMs) were used to analyze density of elephant seals per habitat type, in relation to molt season and weather index as fixed effects, with date and year as random effects (*N* = 90). Models were fitted with a binomial distribution, appropriate for two by two comparisons. As GPS data were repeated measures on the same individual, a second set of GLMMs was used to analyze individual distances moved per day, with explanatory variables of molt stage, windchill, and solar radiation as fixed effects, with date, year, and individual identity as random effects (*N* = 148). In this case, models were fitted with a Poisson distribution, appropriate for count data. Final GLMMs were selected based on the lowest AIC value criteria.

Results were expressed as mean ± standard deviation, unless otherwise specified, and median with interquartile range (i.e., median [quartiles Q_1_; Q_3_]). All statistical analyses were performed with R statistical software (The R Development Core Team, version 3.3.2; RStudio Inc., version 1.0.44).

## 3 RESULTS

### 3.1 Density and habitat selection of elephant seals

Counts of elephant seals in the quadrat Q′ revealed that density was on average of 968 ± 463.0 seals/km² (median [interquartile range]: 960 [610; 1180]; *N* = 45 daily counts). Fieldwork dates, and hence, Q′ counts dates, in 2015, were not concomitant with Q′ counts dates in 2014 and 2016 (Table 1). Hence, we analyzed variation in density with time in 2015 separately from other years. In 2015, density increased from 656 seals/km² to 1600 seals/km² between 25 December and 11 January (nonparametric Spearman's rank correlation: *N* = 11, *r* = .88, *p *=* *.0006). In 2014 and 2016, density decreased from 2224 to 228 seals/km² between 23 January and 28 February (nonparametric Spearman's rank correlation: *N* = 36, *r* = −.70, *p *<* *.0001). Therefore, based on our observations, we defined early molting season from end of December to mid‐January (25 December to 11 January), the peak of the molting season between mid‐January and early February (23 January to 1 February) and late molting season from mid to end of February (11 February to 28 February).

**Figure 3:**
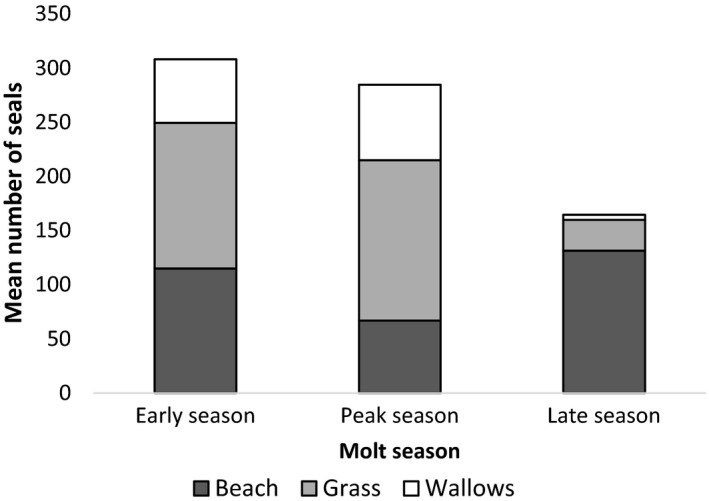
Mean number of female southern elephant seals per habitat during the molting season (data from Q′ counts, from 2014 to 2016)

Counts of elephant seals in the quadrat Q′ allowed us to compare density between different habitat types. In quadrat Q′, use of wallow and nonwallow habitats varied depending on the season. In wallows, there were more seals during the early season (estimate ± *SD* = 2.34 ± 0.57, z = 4.133, *p* < .0001) and during the peak of the season (estimate ± *SD* = 2.53 ± 0.34, *z* = 7.516, *p *<* *.0001; Figure 3) than in the late season. For nonwallow habitats, elephant seals were observed more on grass during the early season (estimate ± *SD* = −5.25 ± 1.78, *z* = −2.954, *p *=* *.0003) and the peak of the season (estimate ± *SD* = −5.45 ± 1.13, *z* = 4.827, *p *<* *.0001), while they were observed more on the beach during the late season (Figure 3).

**Figure 4:**
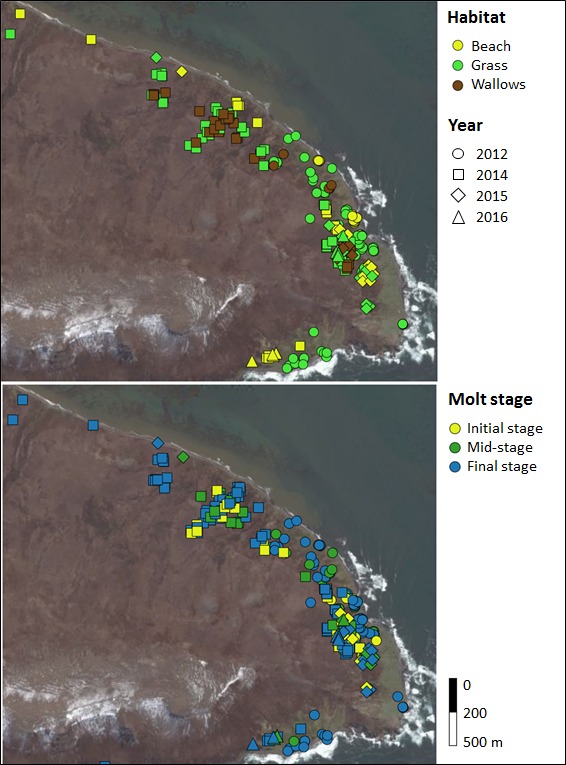
Positions and habitat use of molting female southern elephant seals from visual observations (60 individuals tracked for 3–20 days in 2012, 2014–16)

Habitat use also varied depending on the molt process. We compared stages of molt of elephant seals observed on grass, beach, and wallows (transects T1, T2 and quadrat Q). We observed more seals at initial and mid‐stage of their molt in wallows compared to nonwallow habitats, and more seals at the final stage of molt in nonwallows compared to wallows (nonparametric Kolmogorov–Smirnov test: grass: *D* = 0.59, *p* < .0001; beach: *D* = 0.65, *p *<* *.0001). We also found a difference in the distribution of seals with different molt stages between grass and beach (nonparametric Kolmogorov–Smirnov test: *D* = 0.057, *p *=* *.0002). The majority of seals at the final stage of their molt were on both nonwallow habitats; however, seals at initial or mid‐stage of their molt were observed more on grass than on beach habitat (Figure 4).

**Figure 5:**
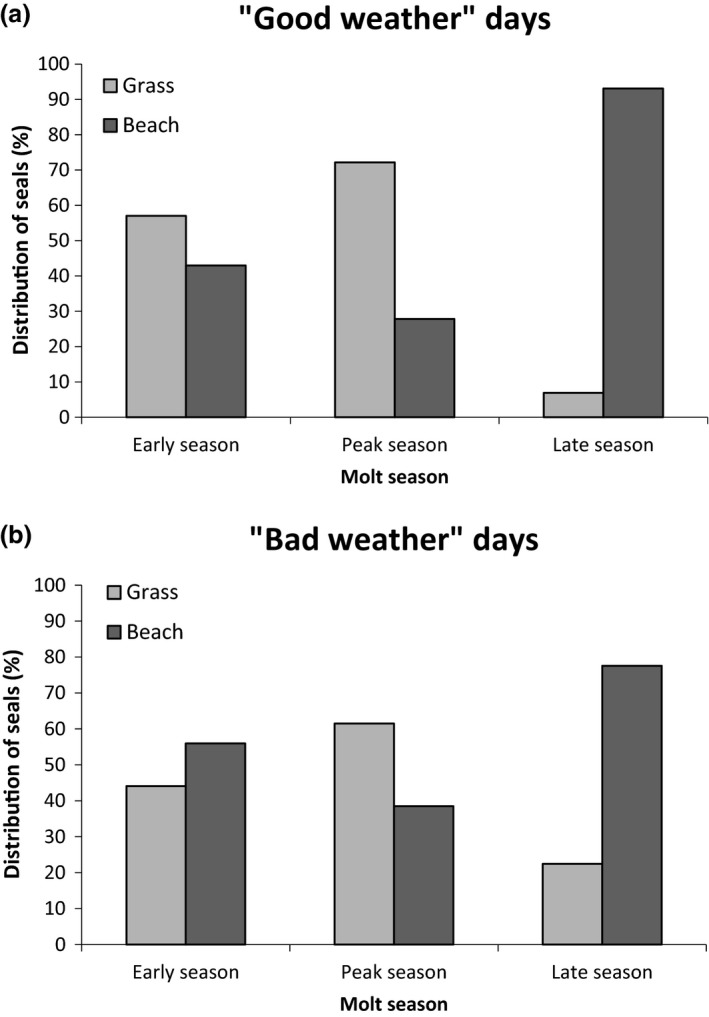
Distribution of female southern elephant seals on nonwallow habitats on “good weather” (a) and “bad weather” (b) days, depending on molt season (data from Q′ counts, from 2014 to 2016)

Variation in habitat use (density per habitat in Q′) was influenced by weather and the molt season (early, peak, and late). There was no relationship between wallow selection and weather index (estimate ± *SD* = 0.03 ± 0.58, *z* = −0.05, *p* = .96). In contrast, nonwallow habitat selection was related to weather index (Figure 5). The model indicated that elephant seals were relatively more abundant on grass during “good weather” days and more abundant on beach habitat during “bad weather” days (estimate ± *SD* = 2.00 ± 0.94, *z* = 2.124, *p *=* *.03). The model that best explained nonwallow habitat selection retained weather index and the molt season as explanatory variables. This indicated that elephant seals tended to select grass over beach during “good weather” days in the early season (estimate ± *SD* = 2.73 ± 1.52, z = 1.79, *p *=* *.07). The model estimated that grass was selected in early season (estimate ± *SD* = 3.74 ± 1.85, *z* = 2.03, *p *=* *.04) and during the peak of the season (estimate ± *SD* = 3.93 ± 1.19, *z* = 3.30, *p *=* *.001) and beach was selected during the late season with no influence of weather (Figure 5).

### 3.2 Movements during the molt

While molting, females occupied both northern and southern coasts of Pointe Suzanne and showed individual differences in movement patterns. Based on movement speed and topography of the study site, we observed that some females stayed in the same restricted area, while others travelled further on land or at sea. In particular, movements at sea were detected for two females in 2012 and 2014 that travelled to Pointe Morne (49°22′S/70°26′E; Figure 2a). They made this return trip of 5000 m at the initial stage (10–40%) and at the final stage of their molt (90–100%) and stayed for several hours (at the initial stage of molt) to three days (at the final stage of molt) at Pointe Morne before returning to Pointe Suzanne to complete the molt. Supposed movements at sea (i.e., movements with speed > 1000 m/h, for GPS locations on the coast) accounted for 0.7% of all movements (*N* = 9 individuals). In order to compare interindividual distances per day with environmental factors that took place on land, we chose to take into account movements on land only for further analyses.

**Figure 6:**
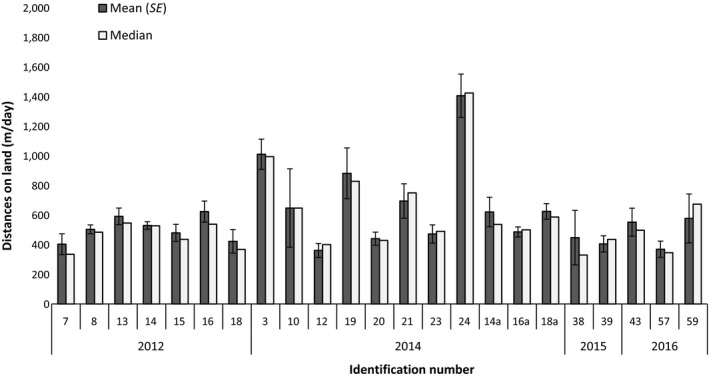
Distances moved by female southern elephant seals on land (m/day) in 2012 and 2014–16 during 6.5 ± 2.75 days

Distances moved by female elephant seals on land averaged 590 ± 237.0 m/day (range 362–1406 m/day; median [interquartile range]: 570 [402; 683]), and there were differences between individuals (nonparametric Kruskal–Wallis rank‐sum test: χ² = 58.17, *df* = 22, *p* = < .0001; Figure 6).

The distance moved by females on land was found to be related to molt stage and weather. Indeed, distances moved per day at mid‐stage (estimate ± *SD* = −0.90 ± 0.14, *z* = −6.63, *p *<* *.0001) or final stage of molt (estimate ± *SD* = −1.45 ± 0.14, *z* = 10.10, *p *<* *.0001) were lower compared to the initial stage. There was no overall effect of solar radiation on individual distances moved (estimate ± *SD* = 0.0002 ± 0.0009, *z* = 0.192, *p *=* *.85). However, distance moved was greater when windchill was low (estimate ± *SD* = −0.25 ± 0.05, *z* = −5.12, *p* < .0001). The model that best explained variation in distances moved during the molt retained both molt stage and meteorological conditions. This indicated that individual distances per day increased when windchill was low, only at mid‐stage (estimate ± *SD* = 0.12 ± 0.02, z = 5.53, *p *<* *.0001) or final stage of molt (estimate ± *SD* = 0.20 ± 0.03, *z* = 7.95, *p* < .0001). The model also showed a significant but weak effect of solar radiation on distances depending on molt stage: distances per day at the final stage increased when solar radiation was high (estimate ± *SD* = 0.0007 ± 0.0003, *z* = 2.55, *p* = .01).

We found no correlation between distance moved by females per day with body mass loss (kg/day) during the molt (nonparametric Spearman's rank correlation: *N* = 19, *r* = −.04, *p *=* *.88). Mean distances moved tended to be negatively correlated with initial mass at capture but this was nonsignificant (nonparametric Spearman's rank correlation: *N* = 22, *r* = −.40, *p *=* *.07). However, there was a negative correlation between individual distances moved and BMIi (nonparametric Spearman's rank correlation: *N* = 22, *r* = −.59, *p* = .05). We also observed a negative correlation between BMIi and the frequency of use of grass habitats (nonparametric Spearman's rank correlation: *N* = 41, *r* = −.45, *p *=* *.003), the opposite effect with the frequency of beach use (nonparametric Spearman's rank correlation: *N* = 41, *r* = .34, *p* = .03), and no correlation was found with the frequency of wallow use (nonparametric Spearman's rank correlation: *N* = 41, *r* = .04, *p* = .80). A positive correlation was found between molt rate and frequency of wallow use (nonparametric Spearman's rank correlation: *N* = 39, *r* = .48, *p *=* *.002), the opposite effect with the frequency of beach use (nonparametric Spearman's rank correlation: *N* = 39, *r* = −.41, *p* = .01), and no correlation with the frequency of grass use (nonparametric Spearman's rank correlation: *N* = 39, *r* = .006, *p* = .97). Aggregation rate (%) tended to be negatively correlated with mean distances moved per day on land (nonparametric Spearman's rank correlation: *N* = 17, *r* = −.45, *p* = .07).

## 4 DISCUSSION

### 4.1 Habitat selection and movements during the molt

The selection of wallows by females during initial and mid‐stage of molt, independent of local weather conditions, suggests that wallow use is strongly linked to the molting process, as previously noted (Boyd et al., 1993; Laws, 1956). During initial and mid‐stages of the molt, elephant seals are most prone to heat loss due to blood perfused bare skin exposed to ambient air (Paterson et al., 2012). We found a positive correlation between the rate of molt and the use of wallows. Individuals seen more often in wallows shed skin/fur more rapidly each day. We suggest two hypotheses to explain the apparent advantage of molting in wallows. Firstly, individuals would have an accelerated loss of old skin and hair due to the mechanical action of body scratching, as elephant seals move against each other in the middle of aggregations (Cruwys & Davis, 1995; Laws, 1956). Secondly, we suggest that wallows where seals closely aggregate would offer a favorable microclimate when exposed to solar radiation. Indeed, temperature measurements indicate that wallows are 4.5°C warmer than air (Chaise et al., in preparation) . This microclimate in a cold sub‐Antarctic climate is favorable to molting: Firstly, because it minimizes heat loss (i.e., energy savings by local microclimate warming; Gilbert et al., 2010) and secondly it may allow the skin to reach an optimal temperature for epidermal cell growth (Feltz & Fay, 1966). Further analyses may confirm these hypotheses (Chaise et al., in preparation). We showed that wallow use was not influenced by weather conditions. This could be due to the fact that wallows represent an optimum but limited resource (Setsaas, Bester, Van Niekerk, Roux, & Hofmeyr, 2008), perhaps due to the topography of the colony site. Hence, skin removal would be the primary driver for wallow use and the thermal advantage of aggregation would be a secondary consequence of this habitat selection.

The two approaches used in our study, animal density counts per habitat and individual molt stage observations along transects, show that the visible molt process may be described in three phases, linked to different habitats. At the initial stage of molt, there appears to be a “search phase” for appropriate wallows to molt, which results in an increase in movements, as found in our study. Hence, at initial and mid‐stages of molt (i.e., early season and peak season), wallows and grass are preferentially used. At the final molting stage (i.e., late molting season with fewer individuals on the colony), individuals were found mainly on the beach and on grass. We suggest that grass is a transition habitat, while seals migrate from beach sites to wallows after their arrival on land and migrate from wallows to beach at the final stage of their molt. We could also suppose that tidal cycles may affect the availability of beach habitat. Further study on movements of elephant seals linked to the variations of the foreshore area during the molt could explore this relationship. Movements were reduced during the mid and final stages of the molt. A decrease in physical activity (i.e., movements) during the main part of the molt would result in energy savings.

Our study is the first to investigate fine‐scale movements of female elephant seals while molting. We showed that distance covered is around 362–1406 m/day. Therefore, elephant seals move more while molting on land than previously thought (Boyd et al., 1993; Laws, 1956). Moreover, we recorded individuals going to sea in 2012 and 2014. These movements occurred at the initial and final stages of their molt and more than 24 h after the day of capture. Our results suggest a more variable pattern of behavior of elephant seals while molting than previously described (Boyd et al., 1993; Setsaas et al., 2008).

### 4.2 Influence of weather conditions

Weather conditions influenced habitat selection for nonwallow habitats (grass and beach). Several studies have described seals entering water during hot days in order to cool while on land, mainly during the breeding season (Codde et al., 2016; White & Odell, 1971; Whittow, 1987). In contrast, our results suggest that, during the molt, seals select grass over beach during “good weather” days. This difference could be due to the fact that the humid surface of the grass represented a cooler environment during warm and sunny days compared to the rocky shore (Chaise et al., in preparation). Moreover, habitat selection of elephant seals may be linked to haul‐out topography. Indeed, Cruwys and Davis (1994) described that only wallows which offered wind protection were selected by elephant seals. At the Pointe Suzanne study site, grass habitat is situated on hills, separated from beach by a few metres of steep gradient (cliff). Therefore, beach selection during “bad weather” days may depend on beach orientation and wind direction (Laws, 1956): Beach could offer a better shelter than the exposed grass surface. Further studies could confirm these findings. The selection of grass during the peak of the season, at mid‐stage of molt, could be related to access to wallows. The selection of beach in late season at the final stage of molt is likely due to their imminent departure to sea (Boyd et al., 1993) or a need to access sea water to drink (Redman et al., 2001).

Our results may suggest that molting elephant seals increased their movements in response to temperature sensation (i.e., windchill), in order to change habitat, as different habitats (topography, substrate) offer different thermal environments (Cruwys & Davis, 1995; Twiss et al., 2002), or to join an aggregation if they were not aggregated before (Cruwys & Davis, 1994). Indeed, we also found a tendency for elephant seals to decrease their distances moved per day when they were aggregated in groups. Our model also estimated an increase in individual distances moved each day when solar radiation was high at the final stage of molt. Despite effects of windchill, high solar radiation may therefore have influenced thermal comfort of elephant seals, leading animals to seek water for thermoregulation (Codde et al., 2016; Cruwys & Davis, 1995; White & Odell, 1971). The fact that distances moved on land were influenced by weather conditions only for elephant seals at mid‐ to final stages of their molt may be due to the fact that location of suitable habitat for molting may have dominated movement patterns, rather than weather constraints. Alternatively, an overall decrease in insulation from the loss of old fur and decrease in blubber thickness may have made animals more sensitive to weather variations (White & Odell, 1971; Worthy et al., 1992).

### 4.3 Body condition and individual strategies

Individual movements on land during the molt approximated 600 m/day, a greater level of activity than previously noted for molting females (Boyd et al., 1993; Laws, 1956). Movement on land is energetically costly (Twiss, Caudron, Pomeroy, Thomas, & Mills, 2000), but we did not find evidence of relatively greater mass loss associated with increased movements.

Elephant seals may adapt their behavior to balance their energetic expenditure (skin and fur renewal, movements on land, thermoregulatory costs), resulting in individual strategies. For example, larger females have relatively less thermoregulatory costs than smaller females (with a higher surface‐area‐to‐volume ratio; Schmidt‐Nielsen, 1997), but energy expenditure may be greater when moving (Schmidt‐Nielsen, 1997). During the molt, elephant seals may maximize energy savings either by decreasing physical activity but with less chance of changing habitat to reduce heat loss, or decreasing heat loss by moving habitat depending on weather conditions. Indeed, females with higher BMI were less active (negative correlation between initial body mass and initial BMI, and mean distances moved per day). Based on these results, we suggest that during the molt females with a higher BMI at arrival (i.e., greater thermal inertia and lower surface area:volume ratio) would be less sensitive to environmental conditions, while a female with a lower BMI at arrival may move from site to site when environmental conditions deteriorate in order to minimize cost of thermoregulation. Initial BMI was also inversely correlated with beach and grass habitat selection. Indeed, larger females or females in better condition tended to arrive later in the molting season (early and peak season: initial body mass = 305 ± 42.7 kg and BMIi = 57.6 ± 4.5 kg/m²; late season: initial body mass = 357 ± 73.9 kg and BMIi = 63.1 ± 6.2 kg/m²; Chaise et al. unpublished). In late season, wallows are emptied as most elephant seals at the final stage of molt selected beach sites. Laws (1956) suggested that molt duration in elephant seals appeared to increase with body size. This could be linked to the negative correlation between molt rate and beach use: Individual females spending more time on beach had slower rate of molt. Females that arrive late in the season, when wallows are emptied, would molt mostly on beach sites and benefit less from optimal molting habitat in wallows. These females would hence show an increase in molting duration (showing a slower molting rate), with concomitant higher fasting duration.

In conclusion, movements and habitat use of female elephant seals during their molt appear to show greater variability than previously documented. Aggregative behavior and habitat selection may be linked to energetic constraints faced while molting on land. Further studies that investigate energy expenditure and behavior during the molt may reveal greater detail on individual strategies of female elephant seals during this relatively understudied stage of their lifecycle.

## ACKNOWLEDGEMENTS

The present research project was supported by the Institut Polaire Français Paul‐Emile Victor (IPEV program 1037). We thank the Terres Australes et Antarctiques Françaises for logistic support, Christophe Guinet (CEBC CNRS; IPEV program 109) for facilitating fieldwork, Pierre‐Yves Henry and Isabelle Hardy (UMR 7179 CNRS/MNHN) for assistance in data treatment, and also Malcolm O'Toole, Pauline Vuarin, and Lucas Delalande for valuable field assistance.

## CONFLICT OF INTEREST

The authors declare that they have no conflict of interest.

## AUTHOR CONTRIBUTIONS

CG, AA, DM, SG, and LC conceived the ideas and designed methodology; CT, SG, LC, and WP collected the data; CT, LC, and IP analyzed the data; LC, CG, DM, AA, and MT led the writing of the manuscript.

## DATA ACCESSIBILITY

No database from the manuscript has been made publicly available yet. Data are available upon request.

## DATA ARCHIVING

We intend to archive data from the manuscript in Dryad Digital Repository: https://doi.org/10.5061/dryad.5ds04gg
