# Supplementary material for: Local weather and body condition influence habitat use and movements on land of molting female southern elephant seals (Mirounga leonina)
Source: Ecol Evol. 2018 May 20;8(12):6081–90. doi: 10.1002/ece3.4049 (PMC6024128; doi:10.1002/ece3.4049)

Supporting information

PCA: weather data from Q’ counts (weather index used for analyses of habitat use at a population level)

| **Variable** | **Mean ± SD** | **PC1_1_** | **PC2_1_** |
| --- | --- | --- | --- |
| Air temperature (°C) | 9.9 ± 3.0 | 0.44 | 0.83 |
| Relative humidity (%) | 76 ± 9.0 | -0.75 | 0.004 |
| Solar radiation (W/m²) | 470 ± 382 | 0.85 | 0.06 |
| Mean wind speed (m/s) | 2.6 ± 1.4 | 0.54 | -0.74 |

Visualization of the variables on the factor map (scatter diagram of the correlation circle)


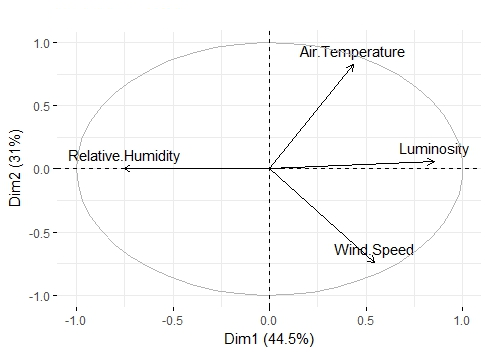

Supplement: Supplementary file 1 [file ECE3-8-6081-s001.docx]
